# Mastocytosis: a mutated KIT receptor induced myeloproliferative disorder

**DOI:** 10.18632/oncotarget.4213

**Published:** 2015-06-05

**Authors:** Anindya Chatterjee, Joydeep Ghosh, Reuben Kapur

**Affiliations:** ^1^ Department of Pediatrics, Herman B Wells Center for Pediatric Research, Indiana University School of Medicine, Indianapolis, Indiana, USA; ^2^ Department of Microbiology and Immunology, Indiana University School of Medicine, Indianapolis, Indiana, USA; ^3^ Department of Medical and Molecular Genetics, Indiana University School of Medicine, Indianapolis, Indiana, USA; ^4^ Department of Molecular Biology and Biochemistry, Indiana University School of Medicine, Indianapolis, Indiana, USA

**Keywords:** mastocytosis, KIT mutations, myeloproliferative disorder, alternative targets in mastocytosis, signaling pathways in mastocytosis

## Abstract

Although more than 90% systemic mastocytosis (SM) patients express gain of function mutations in the KIT receptor, recent next generation sequencing has revealed the presence of several additional genetic and epigenetic mutations in a subset of these patients, which confer poor prognosis and inferior overall survival. A clear understanding of how genetic and epigenetic mutations cooperate in regulating the tremendous heterogeneity observed in these patients will be essential for designing effective treatment strategies for this complex disease. In this review, we describe the clinical heterogeneity observed in patients with mastocytosis, the nature of relatively novel mutations identified in these patients, therapeutic strategies to target molecules downstream from activating KIT receptor and finally we speculate on potential novel strategies to interfere with the function of not only the oncogenic KIT receptor but also epigenetic mutations seen in these patients.

## INTRODUCTION

Based on World Health Organization (WHO) current classification, Myeloproliferative disorders, also known as myeloproliferative neoplasms (MPNs), are grouped into the following seven diseases: (1) Chronic Myelogeneous Leukemia (CML), (2) Chronic Neutrophilic Leukemia (CNL), (3) Polycythemia Vera (PV), (4) Primary Myelofibrosis (PM), (5) Essential Thrombocythemia (ET), (6) Chronic Eosinophilic Leukemia (CEL) and (7) Mastocytosis [1]. These diseases present with a wide range of symptoms. While some of these MPNs progress slowly; others develop into aggressive diseases such as Acute Myeloid Leukemia (AML). Although significant progress has been made in treating some forms of MPNs; Mastocytosis still poses significant challenges. Greater than 90% patients with mastocytosis possess somatic gain-of-function mutations in the KIT receptor tyrosine kinase, primarily an aspartic acid to valine substitution (D816V) in the second catalytic domain, which results in enhanced survival and cell autonomous growth of neoplastic mast cells (MC). Neoplastic mast cells accumulate in different organs, leading to a highly heterogeneous disease, which affects both children and adults. Furthermore, approximately 10–15% oncogenic Kit mutations are also observed in core-binding factor leukemias (CBF-AML) [2–4]. Managing clinical manifestations associated with KIT mutations in mastocytosis has posed significant impediment in large part due to resistance against currently described therapies. In this review, we discuss the challenges and strategies to effectively diagnose, treat, and manage mastocytosis associated with oncogenic KIT and other newly identified mutations in genes encoding epigenetic regulators and spliceosome machinery.

### KIT receptor and mastocytosis

Mast cells (MCs) and their committed progenitors express the KIT receptor (CD117), a Type III receptor tyrosine kinase that is expressed in hematopoietic stem and progenitor cells, germ cells, melanocytes, and interstitial cells of Cajal [5]. The KIT receptor is encoded by a 21-exon containing gene located on human chromosome 4q12, which expresses a 976 amino acid protein with a molecular weight of 145 kDa [6, 7]. The receptor is composed of an extracellular domain (ECD), juxtamembrane domain (JMD), and a tyrosine-kinase domain (TKD). The TKD contains a phosphotransferase domain (PTD) and an ATP binding site. The ligand for KIT receptor, Stem cell factor (SCF) or KIT ligand, induces the development, proliferation, maturation, survival and mediator release from MCs [8]. Under normal physiological conditions, MCs are tightly regulated by the availability of KIT ligand SCF, however under conditions when a gain-of-function receptor is present; uncontrolled proliferation, enhanced survival and cell autonomous growth of MCs can contribute to the disease pathogenesis [9]. The term mastocytosis is attributed to a group of rare heterogeneous disorders that are characterized by abnormal infiltration of neoplastic MCs into one or more organs [10]. Based on its clinical presentation, mastocytosis can be classified into two major forms, cutaneous (CM) and systemic (SM) [1, 10–12]. Mastocytosis affects both children and adults and is mostly acquired, although rare familial cases have been reported [10, 13]. In children, the disease is primarily restricted to the skin and mostly regresses during adolescence. In contrast, in adults the disease can be chronic and systemic in nature. In the pediatric population, the disease predominantly appears in the form of various skin lesions that reveals atypical MC infiltration and aggregates, and is termed as CM. In adults, the disease is chronic and systemic, invariably affecting the bone marrow (BM) and is termed SM [14]. The adult SM patients may manifest the indolent form of the disease or may present with a more aggressive form or even progress to developing leukemic variants of SM with abnormal accumulation of mast cells in various tissues; primarily skin, bone marrow, and visceral organs leading to multi-organ failure and shortened life span [1, 15]. Most adult patients are diagnosed with the indolent form of SM (ISM) where skin is the predominant organ affected, while the aggressive forms of SM (ASM, MC leukemia) are relatively rare in occurrence [10, 13, 16–18]. What is difficult to reconcile is the fact that how a single mutation in the KIT receptor can potentially contribute to such disease heterogeneity. In an effort to explain these observations, recent studies have begun to focus on identifying other non-KIT associated mutations that may contribute to the disease heterogeneity in these patients.

### Prevalence of different KIT mutations in different forms of mastocytosis

In 2008, mastocytosis was diagnosed and classified according to WHO into seven different categories: Cutaneous Mastocytosis (CM), indolent SM (ISM), smoldering SM (SSM), SM with non-associated hematologic non-mast cell disease (SM-AHNMD), aggressive SM (ASM), mast cell leukemia (MCL), MC sarcoma (MCS) and extracutaneous mastocytoma [1, 10, 12, 13, 19–23]. For some of these categories, particularly CM, ISM, SM-AHNMD, ASM and MCL, subvariants have been identified based on observed clinical and biological features (Table 1). WHO guidelines define the diagnosis of SM based on either: the fulfillment of one major criterion along with 1 minor criterion; or fulfillment of at least 3 minor criteria [20, 23]. The major criterion involves multifocal infiltration of MCs (MC burden) in BM or major other organs as determined by tryptase staining. Minor criteria include: (1) presence of atypical MCs in tissues, (2) presence of activating gain-of-function point mutation in KIT D816V in neoplastic MCs in the peripheral blood, BM or visceral organs, (3) aberrant expression of CD2 and/or CD25 in neoplastic MCs, and (4) persistent elevated serum tryptase level (> 20 ng/ml).

**Table 1:** WHO 2008 classification of mastocytosis

Category of Mastocytosis	Abbreviation	Subvariants / subclasses
Cutaneous mastocytosis	CM	**Urticaria pigmentosa (UP)** **Maculopapular CM (MPCM)** **Diffuse CM** **Mastocytoma of skin (MIS)**
Indolent mastocytosis	ISM	**Smoldering SM (SSM)** **Isolated bone marrow mastocytosis** **Well differenciated mastocytosis**
Systemic mastocytosis with associated non-hematological mast cell disease	SM-AHNMD	**SM-acute myeloid leukemia (SM-AML)** **SM-myelodysplastic syndrome (SM-MDS)** **SM-myeloproliferative neoplasm (SM-MPN)** **SM-chronic myelomonocytic leukemia (SM-CMML)** **SM-chronic eosinophilic leukemia (SM-CEL)** **SM-non-Hodgkin lymphoma (SM-NHL)** **SM-myeloma**
Aggressive systemic mastocytosis	ASM	**Lymphadenopathic SM with eosinophilia**
Mast cell leukemia	MCL	**Aleukemic MCL**
Mast cell sarcoma	MCS	
Extracutaneous Mastocytoma	ECM	

*Adapted and complied from* Horny HP, Metcalfe DD, Bennett JM, et al. Mastocytosis. In: Swerdlow SH, Campo E, Harris NL, et al editors. WHO Classification of tumors of haematopoietic and lymphoid tissues. Lyon (France): IARC Press; 2008. p. 54–63, *and from* Bibi S, Langenfeld F, Jeanningros S, Brenet F, Soucie E, Hermine E, Damaj G, Dubreuil P, Arock M, Molecular Defects in Mastocytosis KIT and Beyond KIT, Immunol Allergy Clin N Am 2014. p. 239–262

In adults the presence of KIT D816V mutation is found in > 80% of cases, while in children, *KIT* mutations are found in > 75% of skin biopsies, however only 25% of these are D816V mutations [24–27]. *KIT* mutations in children are primarily localized to the extracellular domain (ECD) and the most frequent mutation reported is a deletion at position 419. The question whether pediatric mastocytosis is a clonal disease also continues to be debated [28, 29]. In general, majority of pediatric patients lack the presence of D816V mutation (only 25–36%) [24, 25], although a significant number of these patients carry additional forms of activating KIT mutations (D835Y, D816I, del417–418, D419Y, C443Y, S476I, ITD502–503, K509I, D572A) [24, 25]. Overall 75% of pediatric patients have some alternations in *KIT*, laying support to the now prevailing hypothesis that childhood mastocytosis is a clonal disease, although the spectrum of the disease and mutations are narrower than those seen in adults [24]. Importantly, in cases of pediatric familial mastocytosis, no KIT D816V mutations have been reported, instead other mutations (K509I, A533D, N822I, S849I, del419, del559–560) and rare germline mutations are observed [30–32] (Table 2). In addition to the spectrum of mutations described above, single nucleotide polymorphisms (SNPs) have also been found in *KIT*, such as (M541L, K546K, I798I, N828N and L862L) [33]. Although the M541L sequence variation seems to confer increased sensitivity to SCF, the clinical significance of this SNP is still unclear.

**Table 2:** Categories of mastocytosis and corresponding type of KIT mutations

Type of Mastocytosis	Prognosis	Treatment options	Type/frequency of KIT and other genetic lesions
Pediatric mastocytosis (PM)	Very Good	Most cases regress with age	**25% KIT WT** **35% KIT D816V/I/Y** **40% ECD KIT mutations**
Indolent mastocytosis (ISM)	Very good to good	No cytoreductive therapy necessary	**> 80% KIT D816V mutations**
Smoldering systemic mastocytosis (SSM)	Relatively good	‘Wait and watch’ in most cases, some may require Interferon (IFN), glucocorticosteriods and cladribine (2CdA)	**> 80% KIT D816V mutations** **Additional non-KIT mutations acquired over disease progression**
Aggressive systemic mastocytosis (ASM)	Poor	IFN, 2CdA; resistant forms treated with TKIs/chemotherapy/hydroxyurea	**> 60% KIT D816V mutations** **Few non-codon 816 KIT mutations** **Additional non-KIT mutations** **(D820G, V559I)**
Systemic mastocytosis with associated non-hematological MC disease (SM-AHNMD)	Depending on the type of SM and prognosis of associated AHNMD	Imatinib to control AHNMD; for SML-AML and aggressive types chemotherapy followed by allogenic stem cell transplantation	**> 80% KIT D816V mutations** **AHNMD presents with genetic lesions** **Non-KIT mutations**
Mast cell leukemia/Mast cell sarcoma (MCL/MCS)	Very poor	Polychemotherapy, allogenic stem cell transplantation, 2CdA, TKIs, Hydroxyurea	**MCL: KIT D816V is least (46%)** **KIT ECD, JMD mutations** **No KIT mutation (WT KIT)** **MCS: No KIT D816V mutation till date (N822K, del419 found)**
Familial mastocytosis	Usually good	Imatinib and TKIs	**Rare somatic KIT D816V mutations** **Germline mutation or deletions (K509I, A533D, R634W, N821I, M835K, S849I or del419, del559-560)**

*Adapted and compiled from* Peter Valent, Mastocytosis: a paradigmatic example of a rare disease with complex biology and pathology, Am J Can Res 2013; 3;159–172; *and from* M. Arock et al, KIT mutation analysis of mast cell neoplasms: recommendations of the European Competence Network on Mastocytosis, Leukemia 2015, 1–10

The presence of activating mutations in adult patients is mostly restricted to the PTD of the KIT receptor [34]. In ISM patients, the presence of KIT D816V is virtually 100%, when detected using sensitive assays on purified BM MCs [35, 36]. A small percentage of ISM cases do progress to an aggressive phenotype, which appears to be determined by the presence of KIT D816V mutation in the non-mast cell compartment acting as a predictor of aggressiveness of the disease. ISM comprises of 2 subsets: well-differentiated SM and SSM (smoldering SM) [11, 13, 23]. Well-differentiated SM is characterized by either non-D816V KIT or absence of KIT mutation. However, SSM is a special subvariant of SM that presents with high mast cell burden, high serum tryptase levels, organomegaly without organ failure but has a clinical course that is stable over many years-hence the name “smoldering”. Some of SSM patients eventually progress to advanced forms of SM (ASM, SM-AHNMD, MCL), while others remain in the smoldering stage. In SSM, the KIT D816V mutation is usually found in the neoplastic MCs, as well as in the non-MC lineage cells [13, 37].

While the prognosis of pediatric CM, ISM and SSM is usually good, in ASM, the prognosis is relatively poor with a median survival of only 41 months [38]. ASM presents itself with progressive evolution leading to impaired BM function, hepatic and splenic failure, fractures and severe weight loss. ASM patients present themselves primarily with KIT D816V mutation in neoplastic MCs, although other *KIT* mutations (D820G, V559I) have also been reported [39, 40] (Table 2). While *KIT* mutations are clearly involved in ASM and its progression to MCL, recent studies demonstrate the presence of additional mutations in these patients, which may help explain the aggressive nature of ASM, progression to MCL and perhaps resistance to tyrosine kinase inhibitors (TKIs) [41–44].

SM-AHNMD occurs between 5–20% of all SM cases and is considered a special subtype of advanced SM. SM-AHNMD is the second most common form of SM and usually occurs in combination with ‘associated clonal hematological non-mast cell lineage disease’ (AHNMD) [45–47]. Although SM-AHNMD is classified as a single disease, it is now known that SM bears a mast cell, while AHNMD bears a myeloid component in most cases (like acute myeloid leukemia (SM-AML) or myeloproliferative neoplasm unclassifiable, chronic myelomonocytic leukemia (SM-CMML), primary myelofibrosis (PMF), atypical chronic myeloid leukemia, myelodysplastic syndrome/myeloproliferative neoplasm unclassificable (SM-MPN), myelodysplastic syndrome (SM-MDS), chronic eosinophilic leukemia (SM-CEL) or non-Hodgkin lymphoma (SM-NHL) [2, 12, 19, 46], (Table 2). In a clinical study comprising of 342 adult SM patients, 94% presented with a KIT mutation (majority bearing D816V mutation) out of which 40% of KIT D816V mutations were present in the AHNMD component [15]. In a separate study comprising of 48 patients with SM-AHNMD were analyzed for the presence of *KIT* mutations in the SM and AHNMD components of the disease; majority of KIT D816V mutations were found in the AHNMD component (89% in SM-CMML and 30% in SM-AML). Interestingly, in these studies, no patients with lymphoproliferative AHNMD displayed *KIT* mutations [46]. Overall, the KIT D816V mutation has been detected in the SM component of all patients, with the exception of CEL, and the frequency of *KIT* mutations in patients bearing AHNMD component is significantly higher than in patients with pure-SM [46]. Presence of KIT D816V mutation in the disease component of SM-AHNMD suggests a clonal relationship between the two components of the disease (SM and AHNMD), with both appearing to have a common precursor (MC/monocytic). One prevailing hypothesis is that KIT D816V is likely to be an early genetic hit in a ‘stem cell’ that gives rise to mast cells (the SM component) and cells of myeloid lineage (the AHNMD component), which likely is followed by a second hit in the AHNMD component, resulting in the development of a more aggressive form of the disease including SM-CMML, SM-MDS and SM-AML [2].

In MCL, the frequency of KIT D816V mutation is less frequent compared to ISM, with only 46% MCL patients expressing this mutation [48]. Interestingly, mutations in the ECD of KIT, juxtamembrane domain (JMD) or complete lack of mutations in the KIT receptor have also been reported in patients with MCL. Thus, although the overall frequency of MCL is rare, the overall prognosis of this disease is dismal. The last subvariant of mastocytosis is called MCS, which is an extremely rare aggressive neoplasm presenting itself as a solitary mass. Till date no KIT D816V mutation has been reported in patients with MCS, although other non-codon *KIT* mutations have been found (N822K, del419) [49] (Table 2). Taken together, the KIT D816V gain-of-function mutation is detected in almost all subtypes of mastocytosis, resulting in uncontrolled activation of the KIT receptor in the appropriate cell type of the BM. However as discussed earlier, the tremendous heterogeneity associated with various forms of mastocytosis cannot be reconciled only by the presence of *KIT* mutations, letting credence to the possible presence of additional genetic abnormalities in these patients. Below we discuss such possibilities.

### Clonal evolution: additional mutations in conjunction with mutated KIT

Although KIT D816V mutations are present in all subtypes of mastocytosis, the presence of the *KIT* mutation does not always correlate with a specific sub-type of SM or its prognosis. Moreover, the KIT D816V mutation is resistant to targeted therapies such as Imatinib (Gleevec), and treatment with newer tyrosine kinase inhibitors (TKIs) like Nilotinib and Dasatinib have shown limited success as many patients relapse after entering remission [50, 51]. Although clinical trials with Midostaurin (PKC412) [41, 52] has yielded promising results, the existing discrepancy of TKIs toward KIT D816V mutations *in vitro* (effective) and *in vivo* (not effective) suggests the possibility of yet unknown additional co-operating mutations that may contribute to the development and pathogenesis of various subtypes of mastocytosis and influence the response to TKI therapy [42, 53, 54]. One such molecular aberration was detected in the tumor suppressor gene *TET2*. In a clinical study involving 48 SM patients, presence of *TET2* mutation was detected in approximately 29% cases [42]. Specifically, *TET2* mutations were identified in 15% of the ISM cases, 40% ASM and 35% of SM-AHNMD patients. Further, 50% of these patients demonstrated the presence of both KIT D816V and *TET2* mutations, suggesting that the high frequency of cooperating (*TET2* – KIT D816V) mutations may contribute to the heterogeneity observed in SM patients. In a separate study, prevalence of *TET2* mutations in mastocytosis patients was observed in 23% patients out of a total number of 26 (15 ISM, 8 SM-AHNMD, 2 ASM, 1 MCS) [53]. Importantly, this same study revealed the presence of *DNMT3A*, *ASXL1* and *CBL* mutations in 12%, 12% and 4%, respectively [53]. Similar to the previous study [42], in this study, 62% of *TET2* mutations were reported in SM-AHNMD sub-group, while only 7% were detected in non-aggressive form of SM, ISM [53]. Interestingly, the SM-AHNMD sub-group co-expressed a combination of more than one genetic and/or epigenetic mutation (e.g. KIT D816V / *TET2* / *DNMT3A* or *TET2 / DNMT3A* or *TET2 / ASLXL1*). Poor overall survival (OS) was observed in patients bearing *TET2* mutations alone, and also in those patients in which more than one mutation was identified including *TET2*, *DNMT3A* and/or *ASXL1*, independent of the oncogenic KIT status. In yet another study involving 74 SM patients, 82.5% patients presented with *KIT* mutations out of which 78.4% showed the presence of KIT D816V mutation [55]. *TET2* deletion was detected in 20.3% of these patients, and all *TET2* mutations co-occurred with KIT D816V mutation, indicating that deregulation of epigenetic genes such as *TET2* could promote the development of severe forms of SM in conjunction with KIT D816V mutations. A more recent study [56] highlighted the presence of additional mutations in a cohort of 62 patients with SM-AHNMD including the presence of *KIT* mutations (87%), *TET2* mutations (27%), *ASXL1* mutations (14%) and CBL mutations (11%). Similar to previously reported clinical findings [42, 53], presence of the KIT D816V mutation significantly correlated with the presence of *TET2* mutations, but not with *ASXL1* mutations. Although the percentage of *TET2* mutations (27%) reported in these studies was higher than *ASXL1* mutations (14%), only the presence of *ASXL1* mutation remained an independent prognostic factor that negatively affected the OS of SM-AHNMD patients [56]. Taken together, these studies suggest that cooperation between *KIT* and *TET2* mutations is likely to contribute to the pathogenesis of mastocytosis; the possibility that *ASXL1* also plays a role in this process cannot be ruled out. The extent to which these mutations function together can only be tested experimentally. In this regard, De Vita *et al*. [57] used a mouse model harboring *TET2* deletion with concomitant KIT D814V expression, and demonstrated that KIT D814V and *TET2* deletion (KIT D814V, *TET2*−/−) cooperates in the mast cell compartment; resulting in a more aggressive phenotype as assessed by the presence of lesions in skin and the digestive tract [57]. More recently, Schwaab *et al*. [43] utilized next generation sequencing to investigate the presence of additional mutations in 39 SM patients. In addition to the presence of KIT D816V mutation in all patients tested, five additional genes were found to be most frequently mutated including *TET2* (39%), *SRSF2* (36%), *ASXL1* (20%), *RUNX1* (23%) and *CBL* (20%). Importantly 10 out of 39 patients carried more than one *TET2* mutation (two patients had four *TET2* mutations), while one patient carried a double mutation in *RUNX1*, and two patients harbored a triple mutation in *CBL*.

In addition to *TET2*, *ASXL1* appears to also be frequently mutated in patients with mastocytosis (12%-20% cases) [43, 53, 56]. Traina and colleagues first reported *ASXL1* as the only other genetic lesion that was detected in a patient with ISM besides KIT D816V, and also in patients with SM-AHNMD. Study by Schwaab *et al*. concluded that the OS of patients with *ASXL1* mutation was significantly shorter than patients harboring the KIT D816V mutation alone. Likewise, Damaj *et al.* showed that the presence of *ASXL1* mutation is an independent prognostic factor that significantly correlates with negative OS. Overall, these clinical findings suggest that the presence of *ASXL1* mutations, besides *KIT* and *TET2* may also contribute to the prognosis and survival of mastocytosis patients. How precisely these mutations contribute to disease pathogenesis remains an active area of investigation.

In addition to the above described mutations, mastocytosis patients also possess mutations in the Spliceosome machinery. Spliceosomes ensure the correct linear order of exons spliced in mRNAs, and mutations in these genes (*SF3B1, SRSF2*, and *U2AF1*) are frequently detected in patients with MDS and CMML [58]. Hanseens *et al*. [59]reported 23.6% *SRSF2*, 5.6% *SF3B1* and 2.7% *U2AF1* mutations in a group of 72 mastocytosis patients and their presence was mutually exclusive. The frequency of *SRSF2* (23.6%) mutations in patients with mastocytosis appeared to be greater than *TET2* mutations, at least in these studies [59]. Importantly, the *SRSF2* mutation was exclusively present in the AHNMD component of all SM-AHNMD patients examined and was specifically identified in the neoplastic MCs and monocytes (i.e. both in SM & AHNMD components). A statistical correlation between the presence of *TET2* and *SRSF2* mutations in this cohort of patients was also observed; however the presence of *SRSF2* mutation did not impact the OS of these patients, unlike that seen in patients with *TET2* mutations. A few studies reported a slightly lower rate of mutations in genes such as *CBL* (3.8%-20.5%), *DNMT3A* (12%), *KRAS, NRAS, JAK2, EZH2* (5.1%) and *ETV6*. *CBL* mutations were mostly found in patients with SM-AHNMD, while *DNMT3A* and *EZH2* mutations were primarily seen in SM patients [43, 53, 54, 56, 59].

With respect to *RAS* mutations (*NRAS, KRAS, HRAS*) in SM; *NRAS* mutation (V12) results in the manifestation of an ASM, MCL and/or SM like disease phenotype in mice. Consistently, wilson and colleagues reported the presence of *NRAS* mutations in 4.5% SM patients in a cohort of 44 patients also bearing the KIT D816V mutation. Interestingly, 2 of these patients demonstrated the presence of *NRAS* mutation but lacked the KIT D816V lesion. Whether *NRAS* mutation precedes *KIT* mutation in the clonal development of mastocytosis remains to be determined [54].

While several clinical studies have shown the co-occurence of genetic and epigenetic mutations in different forms of mastocytosis, a recent study showed that patients with advanced SM could carry as many as 3 (78% of the cases) and in some cases more than 5 mutations (41% of the cases) [43]. The combination of these mutations in these patients varied from the presence of a combination of *KIT-TET2-SRSF2* (26% cases) or *KIT-SRSF2-RUNX1* (18% cases) or *KIT*-*TET2*-*CBL* (13% cases) or *KIT-SRSF2-ASXL1* (10% cases) or *KIT-TETS2-ASXL1* (10% cases). A clinical follow-up was performed on 38 of 39 patients in this study [43]. All 6 patients who died had advanced SM (advSM), but more importantly 5 patients had more than 3 mutations, while 2 patients had more than 5 mutations (besides KIT D816V). These findings highlight the fact that the severity, prognosis and overall survival of patients with advSM might be related to the pattern of mutations that are acquired during the course of the disease. It will be of prime importance to pursue future studies directed at analyzing which group of concurrent mutations (e.g. *KIT*-*TET2*-*ASXL1*), and their sequential onset in the course of disease evolution (e.g. *KIT* followed by *TET2* followed by *ASXL1*) plays a role in establishing the severity of mastocytosis and survival of patients. Gerbaulet *et al*. [60] described a mouse model where expression of KIT D814V mutation in immature hematopoietic cells (stem cells) and mature mast cells gives rise to key pathological features of mastocytosis observed in human patients. This model can be utilized to analyze the role of lesions in respective genes like *KIT, TET2, DNMT3A, ASXL1* and *CBL* in the mast cell compartment, and analyze their relative contribution to the development of advSM. As currently recommended by the European Competence Network on Mastocytosis (ECNM), robust and sensitive methods like ASO-qPCR, RFLP, Nested RT-PCR, NGS should be incorporated to detect various KIT and associated mutations in neoplastic MCs [33]. It will be interesting to see whether the frequency of some of these mutations increases than current reported levels once these diagnostic methods are employed, and/or yet unidentified new mutations are observed in patients with mastocytosis. A summary of mutations described in this section is depicted in Table 3.

**Table 3:** Mutations in KIT D816V, epigenetic regulators and other molecules detected in Mastocytosis

No. of Patients	KIT D816V mutations	TET2 mutations	ASXL1 mutations	DNMT3A mutations	SRSF2 mutations	CBL mutations	EZH2 mutations	RAS mutations	RUNX1 mutations	JAK mutations	Reference
***n* = 48**	20%	29%	*N.D.*	*N.D.*	*N.D.*	*N.D.*	*N.D.*	*N.D.*	*N.D.*	*N.D.*	Tefferi et al.^42^
***n* = 26**	38.4%	23%	12%	11.5%	*N.D.*	3.8%	0%	*N.D.*	*N.D.*	*N.D.*	Traina et al.^53^
***n* = 74**	78.4%	20.3%	*N.D.*	*N.D.*	*N.D.*	*N.D.*	*N.D.*	*N.D.*	*N.D.*	*N.D.*	Soucie et al.^55^
***n* = 62**	87%	27%	14%	*N.D.*	*N.D.*	11%	*N.D.*	*N.D.*	*N.D.*	13%	Damaj et al.^56^
***n* = 39**	100%	39%	20%	*N.D.*	35.8%	20%	5.1%	15.3%	23%	5.1%	Schwaab et al.^43^
***n* = 72**	81%	21%	*N.D.*	12%	23.6%	*N.D*.	*N.D.*	*N.D.*	*N.D.*	*N.D.*	Hanssens et al.^59^
***n* = 44**	100%	*N.D.*	*N.D.*	*N.D.*	*N.D.*	*N.D.*	*N.D.*	4.5%	*N.D.*	*N.D.*	Wilson et al.^54^

Less frequent Mutations in *U2AF1* (5.1%), *ETV6* (2.5%), *SETBP1* (2.5%) have been reported by Schwaab et. al; and mutations in *SF3BP1* (5.6%) and *U2AF1* (2.7%) have been reported by Hanssens et.al. Mutations in *IDH1, IDH2* have not been detected in studies reported thus far. RAS mutations denote total mutations identified in both *KRAS* and *NRAS. N.D.* signifies “Not Determined”. NOTE: Mutations represented above includes total (%) of those present alone (ex. KIT D816V), and with other mutations (ex. KIT D816V + TET2).

### Alternate therapeutic strategy: targeting downstream signaling pathways from the mutated KIT receptor

The mechanism of constitutive activation of the KIT receptor bearing the D816V mutation is at best speculative at present, given that the crystal structure of the kinase domain of KIT has not been identified. It is believed that the D816V mutation results in conformational changes in the phosphotransferase domain (PD) of KIT receptor relieving its auto inhibited state leading to sustained ligand independent activation of the KIT receptor [34]. Several studies have compared the downstream signaling pathways activated *via* the oncogenic KIT D816V versus the WT KIT receptor and have observed both qualitative as well as quantitative differences in their signaling potential. These observations provide rationale to target different signaling molecules that are hijacked by the mutated KIT D816V receptor in neoplastic MCs, thus providing alternative treatment strategies to treat mastocytosis, besides utilizing the currently available options involving TKIs, which show poor responses in clinical trials.

**Figure 1:**
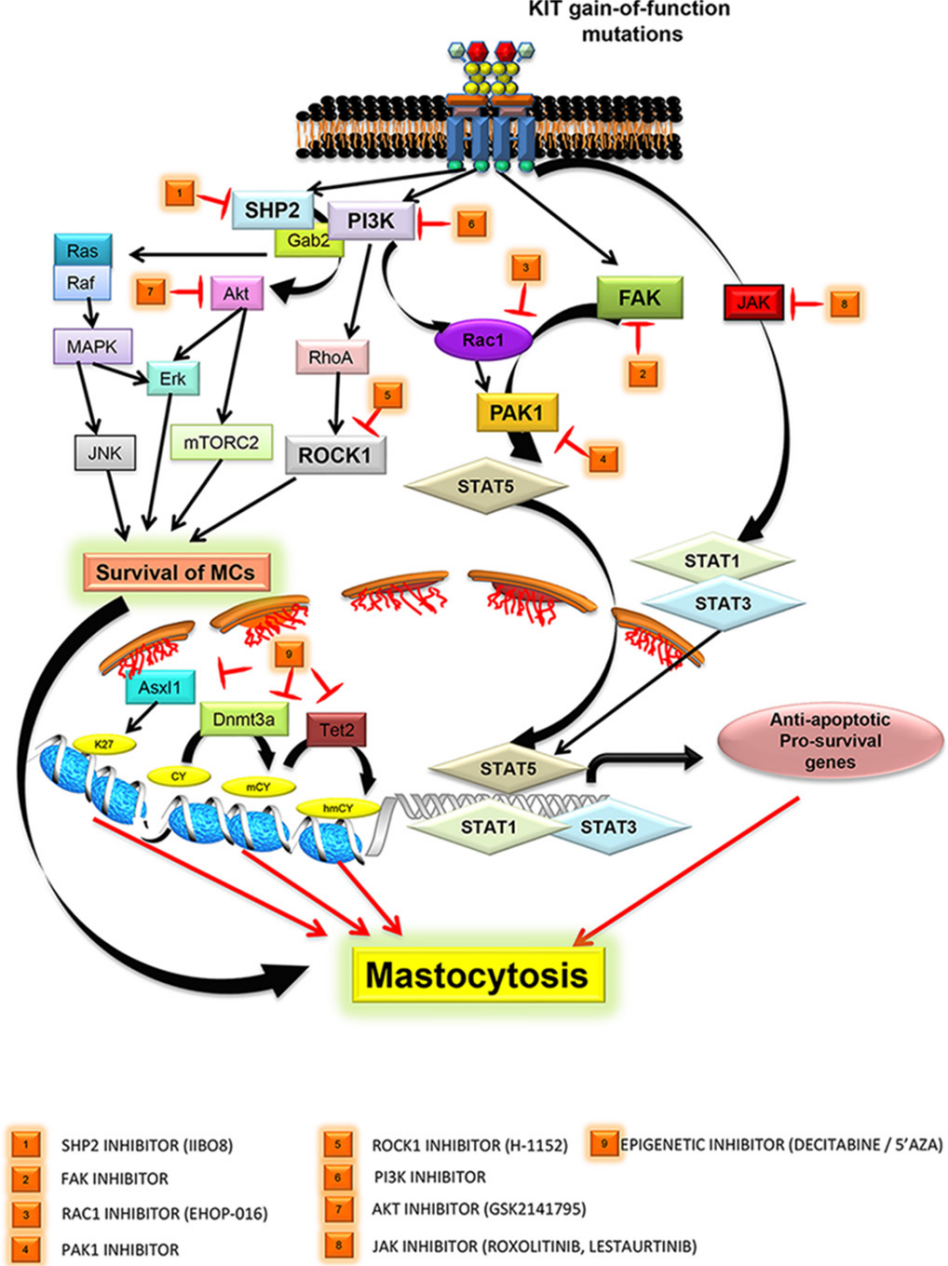
Targeting various downstream signaling pathways from mutated KIT D816V receptors are depicted PI3K mediated activation of RAS-MAPK-JNK pathway, AKT-ERK and ATK-mTORC2 pathway leads to survival of neoplastic MCs. Recent studies highlight newer pathways mediated by SHP2, FAK, ROCK are described with corresponding inhibitors that hold promise. (1) SHP2 and P13K/GAB2 induced AKT/ERK activation can be inhibited using SHP2 specific inhibitor IIBO8, (2) FAK/TIAM1/RAC1/PAK1 mediated nuclear translocation of active STAT5 in SM patients can be inhibited by targeting FAK and PAK1 (4) with inhibitors, (3) PI3K mediated activation of RAC1 *via* VAV1 can be targeted using novel RAC1 inhibitor Ehop-016, (5) PI3K/RHOA mediated activation of ROCK1 can be targeted by inhibitor H-1152 against ROCK1, (6) targeting PI3K using inhibitors that have shown promise in other malignancies [69], (7) targeting AKT (GSK2141795), (8) inhibiting JAK with Roxolitinib, Lestaurtinib, Pacritinib, and (9) targeting epigenetic regulators ASXL1, DNMT3A and TET2 by 5-azacytidine (5′-AZA) and 5-aza-2′ deoxycytidine (Decitabine/DAC).

The subcellular localization of the mutated KIT receptor can trigger unique signals in a cell. To this end, the mutated KIT receptor induces oncogenic signals from the Golgi, and not when localized to the ER, where sustained activation of STAT5 but not of ERK1/2 pathway is observed. Similarly, cell surface localized KIT D816V receptor triggers sustained activation of c-CBL and SHC pathways, however activation of AKT and ERK1/2 requires the availability of SCF (in spite of D816V mutation). Thus, KIT can trigger differential signals based on its subcellular localization and/or its substrate specificity [61–63]. Here, we discuss various signaling pathways that play a role in the transformation of neoplastic MCs (Figure 1). Gleixner and colleagues reported the activation of SRC family kinases (SFKs), LYN and BTK in neoplastic MCs independent of the presence of KIT D816V mutation [64]. Importantly, Dasatinib and Bosutinib deactivated LYN/BTK and rescued neoplastic growth in MCs and a synergistic dose dependent effect on growth repression was observed when Dasatinib was combined with Midostaurin, highlighting the potential of developing new combination therapies to treat mastocytosis. Recently, the mTORC complex components (mTORC 1 & 2) have been attributed in KIT D816V induced neoplastic growth. mTORC1 contributes to MC survival, while mTORC2 plays a critical balance between neoplastic and dividing immature MCs leading to the conclusion that perhaps targeting mTORC2 would reduce the neoplastic MC burden, while leaving the normal MCs unaffected [65]. Upstream from mTORC, AKT also contributes to KIT D816V dependent growth of neoplastic MCs. Active AKT is readily detected in neoplastic MCs obtained from mastocytosis patients, and in human mastocytosis patient derived cell line HMC1.2 active AKT regulates downstream targets like FOXO, NF-kB, and CREB, resulting in anti-apoptotic and sustained cell survival signals that can contribute to malignant growth of neoplastic MCs [32, 66, 67]. Amongst various AKT inhibitors, GSK2141795 has shown some promise in clinical trials [68].

The role of PI3K in various other cancers, besides mastocytosis, is well documented and several agents targeting PI3K are in clinical trials [69]. Similarly, the role of BTK in various other malignancies like Chronic Lymphocytic Leukemia (CLL), Mantle Cell Lymphoma (MCL) and Waldenstrom's Macroglobulinemia (WM) is well established and clinical trials using Ibrutinib [70] has shown favorable outcomes. Given the relative success of PI3K [69] and BTK [70] inhibitors in treating other malignancies, it remains to be seen whether they demonstrate similar efficacy in mastocytosis patients.

In addition to a direct role for the PI3Kinase/AKT pathway, STAT5 is also involved in the growth of neoplastic MCs. Knockdown of STAT5 inhibits the growth of neoplastic MCs. In neoplastic MCs, activated form of STAT5 (pSTAT5) is present in both the nuclear and cytosolic compartments. The presence of pSTAT5 in the cytosol of neoplastic MCs is due to STAT5 association/docking with p85 subunit of PI3K *via* the GAB2 protein. This type of retention of pSTAT5 in the cytosol is attributed to the regulation of AKT signaling pathway in neoplastic MCs. Thus STAT5 function in neoplastic MCs is linked to the PI3K/AKT signaling pathways. Although these studies [66, 71, 72] indicate STAT5 as a bonafide target for treating mastocytosis, designing STAT5 inhibitors that can be used to treat different forms of mastocytosis still remains a challenge and an actively studied area of research. Chaix and colleagues [73] reported a role of JAK in phosphorylation of STAT proteins downstream of KIT D816V mutant bearing cells, and mutations in JAKs (5.1%–13%) [43, 56] have been identified in mastocytosis patients. Together these data indicate that JAKs can also be potential targets to treat mastocytosis. On the other hand, JAK mutations have also been found in CML, and a first generation JAK inhibitor Roxolitinib has been approved to treat myelofibrosis. Second generation JAK inhibitors like Lestaurtinib (CEP-701), and Pacritinib (SB1518) have shown promising results in clinical trials [74]. It remains to be seen whether these inhibitors can also be used for treating patients with mastocytosis.

SHP2 phosphatase is hyperactive and overexpressed in cells bearing the KIT D816V mutation [75]. Using a salicyclic acid based inhibitor II-BO8, which has enhanced selectivity and affinity for SHP2, its activity downstream of KIT D816V can be inhibited, which results in the repression of constitutive growth and survival of these cells. In these cells, constitutively active SHP2 recruits PI3K and GAB2 that leads to the activation of AKT/ERK pathways resulting in KIT D814V mediated transformation. More importantly, treatment of mice transplanted with KIT D814V bearing cells with a combination of II-BO8 and PI3K inhibitor LY294002 shows enhanced survival, than treatment with II-BO8 or LY294002 alone. In another study, downstream of activated KIT D814V receptor, a PI3Kinase/RHOA mediated activation of ROCK1 was shown to induce transformation and myeloproliferative neoplasm in mice [76]. Genetic ablation of ROCK1 or targeting it with a pharmacological inhibitor, H-1152, results in the inhibition of myeloproliferative neoplasm and prolongs the survival of these mice.

More recently, Martin *et al*. [77] demonstrated a VAV1/RAC1/PAK signaling pathway downstream of KIT D814V mutated receptor in triggering myeloproliferative neoplasm in mice. These studies showed that targeting RAC GTPases with a novel small molecule inhibitor Ehop-016 results in a significant repression of disease progression in mice. Mechanistically, treating KIT D814V cells with Ehop-016 results in enhanced cell death *via* repression of an anti-apoptotic protein BAD. Along similar lines, Chatterjee *et al*. [78] demonstrate a novel signaling pathway comprised of FAK/TIAM1/RAC1/PAK1 in regulating the nuclear translocation of active STAT5 with subsequent expression of pro-survival genes resulting in MPN in mice, downstream of oncogenic KIT D814V. Genetic ablation or pharmacological inhibition of FAK or PAK1 resulted in repression of KIT D814V induced MPN development in mice. Moreover in these studies [77, 78], the authors also showed growth repression of neoplastic MCs derived from SM patients that were positive for KIT D816V mutation, when treated with FAK and PAK1 inhibitors, indicating an essential role of FAK and PAK1 in triggering KIT D816V induced SM.

In addition to a role for kinases and phosphatases in regulating KIT D816V induced transformation, a relationship between KIT D816V induced SM and transcription factors like STAT5 and MITF is also evident from recent studies. Hoermann *et al*. [79] demonstrated that in KIT D816V bearing neoplastic mast cells, active STAT5 induces expression of Oncostatin M (OSM), a key modulator of bone marrow microenvironment. Increased OSM expression causes increased angiogenesis, thickened trabeculae and fibrosis; all pathological hallmarks of SM. This study highlighted STAT5 and OSM as potential targets to treat SM [79]. Like STAT5, another transcription factor called microphthalmia-associated transcription factor (MITF) is highly expressed in neoplastic cells derived from bone marrow of SM patients harboring the KIT D816V mutation [80]. At the microRNA (miRNA) level, activating KIT D816V mutation represses the expression of miR-539 and miR-381, which is normally involved in down-regulating MITF in mast cells, demonstrating that KIT D816V promotes the stability of MITF which plays an essential role in proliferative effects seen in mastocytosis. Although the above described signaling pathways are exciting; they will however require, further stringent analysis to determine whether genetic lesions/mutations/sustained activation occur *per se* in RAC1/PAK1/FAK/SHP2 molecules in neoplastic MCs found in mastocytosis patients. Furthermore, a mouse model developed by Gerbaulet *et al*. [60] that shows many cardinal features of human mastocytosis due to the presence of the activating KIT D814V mutation and can be induced to express in multiple hematopoietic stem and progenitor cell lineages (stem cells vs. mast cells), can be further utilized to determine the role of various signaling molecules described above by targeting them using pharmacological inhibitors or genetic ablation, and analyzing their contribution to the development of mastocytosis. These studies will be important to validate SHP2/RAC1/PAK1/FAK as genuine therapeutic targets for treating mastocytosis, along with clinical trials using inhibitors against these molecules.

### Current treatment options and challenges

The majority of patients diagnosed with mastocytosis belong to the indolent category (ISM), a form compatible with an individual's life span. In patients suffering from ISM, a minimum treatment however is necessary, which includes management of symptoms, anti-mediator drugs and careful monitoring of the disease. In contrast, in patients diagnosed with an advanced/aggressive form of SM, which is incurable, and results in a shortened life expectancy; treatment with TKIs has yielded disappointing results. In general, the type of mutation in the KIT receptor determines the efficacy of some of the known TKIs. Patients with KIT mutations in the second catalytic domain (D816V/H/Y/N) tend to demonstrate resistance to Imatinib [81]. This is due to disruption of the conformation of the KIT D816V mutant receptor, rendering the receptor permanently active, which cannot be targeted by Imatinib. On the other hand, treatment with Imatinib has yielded dramatic improvement in MC burden and clinical symptoms in patients with a history of CM harboring a germline mutation in the KIT receptor (F522C). Most mutations found in ECD of the KIT receptor, primarily those detected in pediatric patients, respond well to Imatinib [82, 83]. Likewise, Imatinib treatment of a MCL patient with an ECD KIT mutation (A502-Y503dup) and of patients with no mutations in the KIT receptor (i.e. WT KIT) showed favorable outcomes [84]. Further, several other TKIs have been developed in recent years that can efficiently target the KIT D816V lesion. Dasatinib is potent against cell lines harboring KIT D816V mutations, unfortunately clinical trials yielded disappointing results [51, 64, 85]. Midostaurin (PKC412) is a potent inhibitor of PKC, VEGFR-2, PDGFR, FLT3 and KIT. It inhibits the activity of KIT in HMC mastocytosis cell line, and at pharmacological relevant concentrations against patient derived neoplastic MCs *in vitro*. Consistent with the *in vitro* observations, in several clinical trials, a significant reduction in MC burden in patients with ASM and MCL is observed using Midostaurin [41, 86]. Although initial data from these clinical trials were encouraging, they did not offer long-term remission. Besides TKIs, current non-targeted cytoreductive therapy using Cladribine and Interferon-α have also has shown encouraging results in more aggressive forms of the disease (Table 2).

Another aspect of KIT D816V resistance to current therapies involves the presence of several KIT D816V-independent signaling pathways that are likely to contribute to the survival and abnormal growth of neoplastic MCs [64]. These KIT D816V-independent pathways may support the presence of malignant subclone(s), which may contribute to relapse in patients treated with TKIs who bear KIT D816V negative neoplastic MCs. Thus, therapeutic targeting of additional signaling pathways and molecules may also be a prudent therapeutic approach to treat advanced/aggressive forms of this disease.

One of the major means by which neoplastic mast cells harboring the mutated KIT D816V receptors achieve enhanced survival is through down-regulating the expression of pro-apoptotic and up-regulation of anti-apoptotic proteins. The mutated KIT D816V receptor phosphorylates the pro-apoptotic protein BIM which results in its degradation leading to enhanced survival. The proteasomal inhibitor MG132 up-regulates the expression of BIM and subsequently induces apoptosis in KIT D816V harboring cells, providing a strong rationale for using proteasomal inhibitors as a treatment option for mastocytosis. Obatoclax, an inhibitor of anti-apoptotic family of proteins (BCLXL, BCL2 and MCL1) shows reduced growth and survival in cells expressing KIT D816V mutation. Further, Obatoclax synergizes and potentiates the activity of antineoplastic drugs such as Dasatinib, Midostaurin and Bortezomib, highlighting the importance of combination therapy for SM. Martin *et al*. [77] showed increased apoptosis in KIT D814V bearing cells with a novel RAC1 inhibitor Ehop-016 in part by repressing the expression of anti-apoptotic proteins BAD and MLC, while Chatterjee *et al*. [78] showed that pharmacological targeting of FAK and PAK1 in KIT D816V cells results in down-regulation of anti-apoptotic protein and mRNA levels of BCLXL. A number of FAK inhibitors are currently in clinical trials; whether future clinical studies incorporating FAK, RAC1 and PAK1 inhibitors alone, or in combination with other antineoplastic agents like Dasatinib and Midostaurin provide a better treatment option for ASM patients remains to be seen. Targeting PAK proteins as a therapeutic strategy is currently been tried for various malignancies; as more small molecule inhibitors with increased specificity become available, they are likely to be tested in SM patients. In addition to regulating apoptosis, genetic ablation of PAK1 enhanced the survival of KIT D814V bearing mice in secondary transplants, indicating that targeting PAK1 inhibits the growth/survival of KIT D814V bearing leukemia initiating cell (LIC) [78]. It will be interesting to elucidate the LIC containing fraction in the HSC pool that triggers KIT D814V induced mastocytosis, which subsequently can be targeted using PAK1 inhibitors. This will be an important area of future research, as the LIC population is known to survive conventional therapy, and the survival of LICs has been implicated in the observed relapse often seen in SM patients. Besides molecules described above, clinical trials against BTK, PI3K and JAK have already shown promising results in various other malignancies (discussed earlier under “Alternate Therapeutic Strategy”). Thus targeting BTK, PI3K, JNK in clinical trials involving mastocytosis patients holds significant promise.

In addition to targeting the KIT D816V receptor and its downstream substrates in SM, HMC cells and primary neoplastic mast cells derived from aggressive SM patients carrying the KIT D816V mutation appear to be sensitive to treatment by demethylating agents including 5-azacytidine (5-AZA) and 5-aza-2′deoxycytidine (Decitabine / DAC). 5-AZA and DAC do not alter the expression of KIT receptor, nor do they impact the activation of downstream signaling proteins like AKT, STAT5 or STAT3; rather they induce apoptosis by re-expression of FAS (Fas cell surface death receptor) [87]. Importantly, 5-AZA and DAC do not show any appreciable toxicity toward normal bone marrow or mast cells. Along these lines, a combinatorial approach involving Dasatinib (DASA) and DAC in treating cells derived from SM patients that harbor the KIT D816V and *TET2* deletion was described [57]. Since loss of *TET2* causes aberrant methylation of promoter regions in AML [88], a similar effect in SM patients was predicted. To investigate this hypothesis, the authors knocked down *TET2* in HMC1.2 cells harboring the KIT D816V mutation, and pre-treated cells with low doses of DAC followed by DASA and determined cell death. Loss of *TET2* sensitized SM cells to a combination of drugs (DAC+DASA), leading to significantly higher apoptosis, than in cells treated with each drug alone [57]. This approach paves the way for future treatment strategies for KIT D816V positive and *TET2* mutation carrying SM patients, where pre-treatment with demethylating agents like DAC followed by TKIs like Dasatinib and Midostaurin may yield better therapeutic outcomes. In addition to the above described strategy of using drug combinations, a synergistic inhibitory effect exerted by a combination treatment of Ponatinib and Midostaurin or Dasatinib on the growth of neoplastic mast cells has also been described [52]. Whether results from combination treatments *in vitro* translate into successful clinical studies remains to be seen, as it will take considerable effort to elucidate the sequential order of drugs, and concentrations that do not confer toxicity. Further, it might be valuable to consider a three-pronged therapeutic approach for the treatment of SM patients harboring the KIT D816V mutation. In this scenario, co-targeting downstream effector molecules such as SHP2, FAK, RAC1, and PAK1 along with the KIT D816V receptor itself using TKIs, and epigenetic regulators with demethylating agents might result in better outcomes (Figure 1).

The challenges posed by the mutated KIT D816V receptor has resulted in the development of newer strategies for designing potent inhibitors by exploiting the unique structural conformation KIT receptor adopts in its active versus its inactive state. Bai *et al*. [89] utilized a rational drug design to target the ‘switch pocket’ (SP) region of KIT that is occupied by different switch regions giving the receptor either an ‘active’ or ‘inactive’ conformation. The drugs DP-2976 and DP-4851 exhibit potent anti-neoplastic activity against HMC and bone marrow mast cells derived from SM patients [89]. These drugs hold significant promise since mechanistically they function differently from current drugs like Imatinib and Midostaurin, which are ATP competitors, while DP-2976 and DP-4851 are non-ATP competitors. Lee at al. [90] have reported the development of a new series of 7-azaindole based inhibitors that show a 100-fold increased sensitivity toward the KIT D816V mutation than wild-type KIT receptor. It will be interesting to see results using these drugs in future clinical trials.

### Concluding remarks and future perspectives

In mastocytosis, both in the indolent (ISM) and in the aggressive (ASM) form of the disease, mutations in the KIT receptor (D816V and others) and genetic lesions in various signaling molecules and modifiers are observed. To treat the aggressive form of the disease, targeting the activated version of the mutated KIT receptor with drugs has been largely ineffective, therefore co-targeting selective molecules in signaling pathways downstream of the mutated KIT receptor is likely to be prudent therapeutic strategy. Moreover, application of combination therapies during the initial phase of disease may optimize the clearance of neoplastic MC burden that may in-turn reduce drug-resistance induced relapse. However, to efficiently eradicate neoplastic MCs, targeting tumor stem/leukemia initiating cells (LIC) is also necessary. Importantly, the detection and quantification of KIT allele burden will be critical for monitoring the disease, its prognosis during the course of the disease and during/after therapies. Fortunately, highly specific PCR-based assays are readily available that should be incorporated as a standard routine in screening mastocytosis patients with KIT mutations. Moreover, to expand the breadth of screening for mutations in newly described players in this disease such as *TET2*, *ASXL1* and *SRSF2*; methods such as next-generation sequencing (NGS) and evaluation of myeloid-gene mutation panel are likely to be of benefit [33]. These approaches will hopefully provide the tools to diagnose and treat the mutational patterns, course and aggressiveness of the disease and alleviate the suffering in mastocytosis patients.

## References

[1] Pardanani A (2013). Systemic mastocytosis in adults: 2013 update on diagnosis, risk stratification, and management. American journal of hematology.

[2] Wang SA, Hutchinson L, Tang G, Chen SS, Miron PM, Huh YO, Jones DM, Bueso-Ramos C, Verstovsek S, Medeiros LJ, Miranda RN (2013). Systemic mastocytosis with associated clonal hematological non-mast cell lineage disease: clinical significance and comparison of chomosomal abnormalities in SM and AHNMD components. American journal of hematology.

[3] Nick HJ, Kim HG, Chang CW, Harris KW, Reddy V, Klug CA (2012). Distinct classes of c-Kit-activating mutations differ in their ability to promote RUNX1-ETO-associated acute myeloid leukemia. Blood.

[4] Zhao L, Melenhorst JJ, Alemu L, Kirby M, Anderson S, Kench M, Hoogstraten-Miller S, Brinster L, Kamikubo Y, Gilliland DG, Liu PP (2012). KIT with D816 mutations cooperates with CBFB-MYH11 for leukemogenesis in mice. Blood.

[5] Miettinen M, Lasota J (2005). KIT (CD117): a review on expression in normal and neoplastic tissues, and mutations and their clinicopathologic correlation. Applied immunohistochemistry & molecular morphology : AIMM / official publication of the Society for Applied Immunohistochemistry.

[6] Giebel LB, Strunk KM, Holmes SA, Spritz RA (1992). Organization and nucleotide sequence of the human KIT (mast/stem cell growth factor receptor) proto-oncogene. Oncogene.

[7] Chabot B, Stephenson DA, Chapman VM, Besmer P, Bernstein A (1988). The proto-oncogene c-kit encoding a transmembrane tyrosine kinase receptor maps to the mouse W locus. Nature.

[8] Metcalfe DD (2008). Mast cells and mastocytosis. Blood.

[9] Piao X, Bernstein A (1996). A point mutation in the catalytic domain of c-kit induces growth factor independence, tumorigenicity, and differentiation of mast cells. Blood.

[10] Valent P (2008). Systemic mastocytosis. Cancer Treat Res.

[11] Arock M, Valent P (2010). Pathogenesis, classification and treatment of mastocytosis: state of the art in 2010 and future perspectives. Expert Rev Hematol.

[12] Valent P, Jaffe ES, Harris NL, Stein H (2001). Mastocytosis (mast cell disease). World Health Organization (WHO) classification of tumors. Pathology & genetics. Tumors of haematopoietic and lymphoid tissues.

[13] Valent P (2013). Mastocytosis: a paradigmatic example of a rare disease with complex biology and pathology. Am J Cancer Res.

[14] Feger F, Ribadeau Dumas A, Leriche L, Valent P, Arock M (2002). Kit and c-kit mutations in mastocytosis: a short overview with special reference to novel molecular and diagnostic concepts. Int Arch Allergy Immunol.

[15] Lim KH, Tefferi A, Lasho TL, Finke C, Patnaik M, Butterfield JH, McClure RF, Li CY, Pardanani A (2009). Systemic mastocytosis in 342 consecutive adults: survival studies and prognostic factors. Blood.

[16] Ryan RJ, Akin C, Castells M, Wills M, Selig MK, Nielsen GP, Ferry JA, Hornick JL (2013). Mast cell sarcoma: a rare and potentially under-recognized diagnostic entity with specific therapeutic implications. Mod Pathol.

[17] Briley LD, Phillips CM (2008). Cutaneous mastocytosis: a review focusing on the pediatric population. Clin Pediatr (Phila).

[18] Pardanani A (2012). Systemic mastocytosis: disease overview, pathogenesis, and treatment. Hematol Oncol Clin North Am.

[19] Erben P, Schwaab J, Metzgeroth G, Horny HP, Jawhar M, Sotlar K, Fabarius A, Teichmann M, Schneider S, Ernst T, Muller MC, Giehl M, Marx A, Hartmann K, Hochhaus A, Hofmann WK (2014). The KIT D816V expressed allele burden for diagnosis and disease monitoring of systemic mastocytosis. Annals of hematology.

[20] Valent P, Akin C, Escribano L, Fodinger M, Hartmann K, Brockow K, Castells M, Sperr WR, Kluin-Nelemans HC, Hamdy NA, Lortholary O, Robyn J, van Doormaal J, Sotlar K, Hauswirth AW, Arock M (2007). Standards and standardization in mastocytosis: consensus statements on diagnostics, treatment recommendations and response criteria. Eur J Clin Invest.

[21] Valent P, Akin C, Sperr WR, Mayerhofer M, Fodinger M, Fritsche-Polanz R, Sotlar K, Escribano L, Arock M, Horny HP, Metcalfe DD (2005). Mastocytosis: pathology, genetics, and current options for therapy. Leuk Lymphoma.

[22] Valent P, Horny HP, Bennett JM, Fonatsch C, Germing U, Greenberg P, Haferlach T, Haase D, Kolb HJ, Krieger O, Loken M, van de Loosdrecht A, Ogata K, Orfao A, Pfeilstocker M, Ruter B (2007). Definitions and standards in the diagnosis and treatment of the myelodysplastic syndromes: Consensus statements and report from a working conference. Leuk Res.

[23] Valent P, Horny HP, Escribano L, Longley BJ, Li CY, Schwartz LB, Marone G, Nunez R, Akin C, Sotlar K, Sperr WR, Wolff K, Brunning RD, Parwaresch RM, Austen KF, Lennert K (2001). Diagnostic criteria and classification of mastocytosis: a consensus proposal. Leuk Res.

[24] Bibi S, Langenfeld F, Jeanningros S, Brenet F, Soucie E, Hermine O, Damaj G, Dubreuil P, Arock M (2014). Molecular defects in mastocytosis: KIT and beyond KIT. Immunol Allergy Clin North Am.

[25] Bodemer C, Hermine O, Palmerini F, Yang Y, Grandpeix-Guyodo C, Leventhal PS, Hadj-Rabia S, Nasca L, Georgin-Lavialle S, Cohen-Akenine A, Launay JM, Barete S, Feger F, Arock M, Catteau B, Sans B (2010). Pediatric mastocytosis is a clonal disease associated with D816V and other activating c-KIT mutations. J Invest Dermatol.

[26] Verzijl A, Heide R, Oranje AP, van Schaik RH (2007). C-kit Asp-816-Val mutation analysis in patients with mastocytosis. Dermatology.

[27] Yanagihori H, Oyama N, Nakamura K, Kaneko F (2005). c-kit Mutations in patients with childhood-onset mastocytosis and genotype-phenotype correlation. J Mol Diagn.

[28] Longley BJ, Metcalfe DD, Tharp M, Wang X, Tyrrell L, Lu SZ, Heitjan D, Ma Y (1999). Activating and dominant inactivating c-KIT catalytic domain mutations in distinct clinical forms of human mastocytosis. Proc Natl Acad Sci U S A.

[29] Buttner C, Henz BM, Welker P, Sepp NT, Grabbe J (1998). Identification of activating c-kit mutations in adult-, but not in childhood-onset indolent mastocytosis: a possible explanation for divergent clinical behavior. J Invest Dermatol.

[30] Zhang LY, Smith ML, Schultheis B, Fitzgibbon J, Lister TA, Melo JV, Cross NC, Cavenagh JD (2006). A novel K509I mutation of KIT identified in familial mastocytosis-*in vitro* and *in vivo* responsiveness to imatinib therapy. Leuk Res.

[31] Hoffmann KM, Moser A, Lohse P, Winkler A, Binder B, Sovinz P, Lackner H, Schwinger W, Benesch M, Urban C (2008). Successful treatment of progressive cutaneous mastocytosis with imatinib in a 2-year-old boy carrying a somatic KIT mutation. Blood.

[32] Yang Y, Letard S, Borge L, Chaix A, Hanssens K, Lopez S, Vita M, Finetti P, Birnbaum D, Bertucci F, Gomez S, de Sepulveda P, Dubreuil P (2010). Pediatric mastocytosis-associated KIT extracellular domain mutations exhibit different functional and signaling properties compared with KIT-phosphotransferase domain mutations. Blood.

[33] Arock M, Sotlar K, Akin C, Broesby-Olsen S, Hoermann G, Escribano L, Kristensen TK, Kluin-Nelemans HC, Hermine O, Dubreuil P, Sperr WR, Hartmann K, Gotlib J, Cross NC, Haferlach T, Garcia-Montero A (2015). KIT mutation analysis in mast cell neoplasms: recommendations of the European Competence Network on Mastocytosis. Leukemia.

[34] Laine E, Chauvot de Beauchene I, Perahia D, Auclair C, Tchertanov L (2011). Mutation D816V alters the internal structure and dynamics of c-KIT receptor cytoplasmic region: implications for dimerization and activation mechanisms. PLoS Comput Biol.

[35] Garcia-Montero AC, Jara-Acevedo M, Teodosio C, Sanchez ML, Nunez R, Prados A, Aldanondo I, Sanchez L, Dominguez M, Botana LM, Sanchez-Jimenez F, Sotlar K, Almeida J, Escribano L, Orfao A (2006). KIT mutation in mast cells and other bone marrow hematopoietic cell lineages in systemic mast cell disorders: a prospective study of the Spanish Network on Mastocytosis (REMA) in a series of 113 patients. Blood.

[36] Kristensen T, Vestergaard H, Moller MB (2011). Improved detection of the KIT D816V mutation in patients with systemic mastocytosis using a quantitative and highly sensitive real-time qPCR assay. J Mol Diagn.

[37] Jordan JH, Fritsche-Polanz R, Sperr WR, Mitterbauer G, Fodinger M, Schernthaner GH, Christian Bankl H, Gebhart W, Chott A, Lechner K, Valent P (2001). A case of ‘smouldering’ mastocytosis with high mast cell burden, monoclonal myeloid cells, and C-KIT mutation Asp-816-Val. Leuk Res.

[38] Gotlib J, Pardanani A, Akin C, Reiter A, George T, Hermine O, Kluin-Nelemans H, Hartmann K, Sperr WR, Brockow K, Schwartz LB, Orfao A, Deangelo DJ, Arock M, Sotlar K, Horny HP (2013). International Working Group-Myeloproliferative Neoplasms Research and Treatment (IWG-MRT) & European Competence Network on Mastocytosis (ECNM) consensus response criteria in advanced systemic mastocytosis. Blood.

[39] Pignon JM, Giraudier S, Duquesnoy P, Jouault H, Imbert M, Vainchenker W, Vernant JP, Tulliez M (1997). A new c-kit mutation in a case of aggressive mast cell disease. British journal of haematology.

[40] Nakagomi N, Hirota S (2007). Juxtamembrane-type c-kit gene mutation found in aggressive systemic mastocytosis induces imatinib-resistant constitutive KIT activation. Lab Invest.

[41] Ustun C, DeRemer DL, Akin C (2011). Tyrosine kinase inhibitors in the treatment of systemic mastocytosis. Leuk Res.

[42] Tefferi A, Levine RL, Lim KH, Abdel-Wahab O, Lasho TL, Patel J, Finke CM, Mullally A, Li CY, Pardanani A, Gilliland DG (2009). Frequent TET2 mutations in systemic mastocytosis: clinical, KITD816V and FIP1L1-PDGFRA correlates. Leukemia.

[43] Schwaab J, Schnittger S, Sotlar K, Walz C, Fabarius A, Pfirrmann M, Kohlmann A, Grossmann V, Meggendorfer M, Horny HP, Valent P, Jawhar M, Teichmann M, Metzgeroth G, Erben P, Ernst T (2013). Comprehensive mutational profiling in advanced systemic mastocytosis. Blood.

[44] Valent P (1994). The riddle of the mast cell: kit(CD117)-ligand as the missing link?. Immunol Today.

[45] Valent P, Sperr WR, Akin C (2010). How I treat patients with advanced systemic mastocytosis. Blood.

[46] Sotlar K, Colak S, Bache A, Berezowska S, Krokowski M, Bultmann B, Valent P, Horny HP (2010). Variable presence of KITD816V in clonal haematological non-mast cell lineage diseases associated with systemic mastocytosis (SM-AHNMD). The Journal of pathology.

[47] Pardanani A, Lim KH, Lasho TL, Finke C, McClure RF, Li CY, Tefferi A (2009). Prognostically relevant breakdown of 123 patients with systemic mastocytosis associated with other myeloid malignancies. Blood.

[48] Georgin-Lavialle S, Lhermitte L, Dubreuil P, Chandesris MO, Hermine O, Damaj G (2013). Mast cell leukemia. Blood.

[49] Georgin-Lavialle S, Aguilar C, Guieze R, Lhermitte L, Bruneau J, Fraitag S, Canioni D, Chandesris MO, Suarez F, Grandpeix-Guyodo C, Damaj G, Barete S, Aouba A, Fite C, Robert C, Gaulard P (2013). Mast cell sarcoma: a rare and aggressive entity—report of two cases and review of the literature. J Clin Oncol.

[50] Ma Y, Zeng S, Metcalfe DD, Akin C, Dimitrijevic S, Butterfield JH, McMahon G, Longley BJ (2002). The c-KIT mutation causing human mastocytosis is resistant to STI571 and other KIT kinase inhibitors; kinases with enzymatic site mutations show different inhibitor sensitivity profiles than wild-type kinases and those with regulatory-type mutations. Blood.

[51] Shah NP, Lee FY, Luo R, Jiang Y, Donker M, Akin C (2006). Dasatinib (BMS-354825) inhibits KITD816V, an imatinib-resistant activating mutation that triggers neoplastic growth in most patients with systemic mastocytosis. Blood.

[52] Gleixner KV, Peter B, Blatt K, Suppan V, Reiter A, Radia D, Hadzijusufovic E, Valent P (2013). Synergistic growth-inhibitory effects of ponatinib and midostaurin (PKC412) on neoplastic mast cells carrying KIT D816V. Haematologica.

[53] Traina F, Visconte V, Jankowska AM, Makishima H, O'Keefe CL, Elson P, Han Y, Hsieh FH, Sekeres MA, Mali RS, Kalaycio M, Lichtin AE, Advani AS, Duong HK, Copelan E, Kapur R (2012). Single nucleotide polymorphism array lesions, TET2, DNMT3A, ASXL1 and CBL mutations are present in systemic mastocytosis. PloS one.

[54] Wilson TM, Maric I, Simakova O, Bai Y, Chan EC, Olivares N, Carter M, Maric D, Robyn J, Metcalfe DD (2011). Clonal analysis of NRAS activating mutations in KIT-D816V systemic mastocytosis. Haematologica.

[55] Soucie E, Hanssens K, Mercher T, Georgin-Lavialle S, Damaj G, Livideanu C, Chandesris MO, Acin Y, Letard S, de Sepulveda P, Hermine O, Bernard OA, Dubreuil P (2012). In aggressive forms of mastocytosis, TET2 loss cooperates with c-KITD816V to transform mast cells. Blood.

[56] Damaj G, Joris M, Chandesris O, Hanssens K, Soucie E, Canioni D, Kolb B, Durieu I, Gyan E, Livideanu C, Cheze S, Diouf M, Garidi R, Georgin-Lavialle S, Asnafi V, Lhermitte L (2014). ASXL1 but not TET2 mutations adversely impact overall survival of patients suffering systemic mastocytosis with associated clonal hematologic non-mast-cell diseases. PloS one.

[57] De Vita S, Schneider RK, Garcia M, Wood J, Gavillet M, Ebert BL, Gerbaulet A, Roers A, Levine RL, Mullally A, Williams DA (2014). Loss of function of TET2 cooperates with constitutively active KIT in murine and human models of mastocytosis. PloS one.

[58] Meggendorfer M, Roller A, Haferlach T, Eder C, Dicker F, Grossmann V, Kohlmann A, Alpermann T, Yoshida K, Ogawa S, Koeffler HP, Kern W, Haferlach C, Schnittger S (2012). SRSF2 mutations in 275 cases with chronic myelomonocytic leukemia (CMML). Blood.

[59] Hanssens K, Brenet F, Agopian J, Georgin-Lavialle S, Damaj G, Cabaret L, Chandesris MO, de Sepulveda P, Hermine O, Dubreuil P, Soucie E (2014). SRSF2-p95 hotspot mutation is highly associated with advanced forms of mastocytosis and mutations in epigenetic regulator genes. Haematologica.

[60] Gerbaulet A, Wickenhauser C, Scholten J, Peschke K, Drube S, Horny HP, Kamradt T, Naumann R, Muller W, Krieg T, Waskow C, Hartmann K, Roers A (2011). Mast cell hyperplasia, B-cell malignancy, and intestinal inflammation in mice with conditional expression of a constitutively active kit. Blood.

[61] Xiang Z, Kreisel F, Cain J, Colson A, Tomasson MH (2007). Neoplasia driven by mutant c-KIT is mediated by intracellular, not plasma membrane, receptor signaling. Mol Cell Biol.

[62] Choudhary C, Olsen JV, Brandts C, Cox J, Reddy PN, Bohmer FD, Gerke V, Schmidt-Arras DE, Berdel WE, Muller-Tidow C, Mann M, Serve H (2009). Mislocalized activation of oncogenic RTKs switches downstream signaling outcomes. Mol Cell.

[63] Chian R, Young S, Danilkovitch-Miagkova A, Ronnstrand L, Leonard E, Ferrao P, Ashman L, Linnekin D (2001). Phosphatidylinositol 3 kinase contributes to the transformation of hematopoietic cells by the D816V c-Kit mutant. Blood.

[64] Gleixner KV, Mayerhofer M, Cerny-Reiterer S, Hormann G, Rix U, Bennett KL, Hadzijusufovic E, Meyer RA, Pickl WF, Gotlib J, Horny HP, Reiter A, Mitterbauer-Hohendanner G, Superti-Furga G, Valent P (2011). KIT-D816V-independent oncogenic signaling in neoplastic cells in systemic mastocytosis: role of Lyn and Btk activation and disruption by dasatinib and bosutinib. Blood.

[65] Smrz D, Kim MS, Zhang S, Mock BA, Smrzova S, DuBois W, Simakova O, Maric I, Wilson TM, Metcalfe DD, Gilfillan AM (2011). mTORC1 and mTORC2 differentially regulate homeostasis of neoplastic and non-neoplastic human mast cells. Blood.

[66] Harir N, Boudot C, Friedbichler K, Sonneck K, Kondo R, Martin-Lanneree S, Kenner L, Kerenyi M, Yahiaoui S, Gouilleux-Gruart V, Gondry J, Benit L, Dusanter-Fourt I, Lassoued K, Valent P, Moriggl R (2008). Oncogenic Kit controls neoplastic mast cell growth through a Stat5/PI3-kinase signaling cascade. Blood.

[67] Gabillot-Carre M, Lepelletier Y, Humbert M, de Sepuvelda P, Hamouda NB, Zappulla JP, Liblau R, Ribadeau-Dumas A, Machavoine F, Letard S, Baude C, Hermant A, Yang Y, Vargaftig J, Bodemer C, Morelon E (2006). Rapamycin inhibits growth and survival of D816V-mutated c-kit mast cells. Blood.

[68] Pal SK, Reckamp K, Yu H, Figlin RA (2010). Akt inhibitors in clinical development for the treatment of cancer. Expert Opin Investig Drugs.

[69] Rodon J, Dienstmann R, Serra V, Tabernero J (2013). Development of PI3K inhibitors: lessons learned from early clinical trials. Nat Rev Clin Oncol.

[70] Burger JA (2014). Bruton's tyrosine kinase (BTK) inhibitors in clinical trials. Curr Hematol Malig Rep.

[71] Baumgartner C, Cerny-Reiterer S, Sonneck K, Mayerhofer M, Gleixner KV, Fritz R, Kerenyi M, Boudot C, Gouilleux F, Kornfeld JW, Sillaber C, Moriggl R, Valent P (2009). Expression of activated STAT5 in neoplastic mast cells in systemic mastocytosis: subcellular distribution and role of the transforming oncoprotein KIT D816V. The American journal of pathology.

[72] Morales JK, Falanga YT, Depcrynski A, Fernando J, Ryan JJ (2010). Mast cell homeostasis and the JAK-STAT pathway. Genes Immun.

[73] Chaix A, Lopez S, Voisset E, Gros L, Dubreuil P, De Sepulveda P (2011). Mechanisms of STAT protein activation by oncogenic KIT mutants in neoplastic mast cells. J Biol Chem.

[74] Sonbol MB, Firwana B, Zarzour A, Morad M, Rana V, Tiu RV (2013). Comprehensive review of JAK inhibitors in myeloproliferative neoplasms. Ther Adv Hematol.

[75] Mali RS, Ma P, Zeng LF, Martin H, Ramdas B, He Y, Sims E, Nabinger S, Ghosh J, Sharma N, Munugalavadla V, Chatterjee A, Li S, Sandusky G, Craig AW, Bunting KD (2012). Role of SHP2 phosphatase in KIT-induced transformation: identification of SHP2 as a druggable target in diseases involving oncogenic KIT. Blood.

[76] Mali RS, Ramdas B, Ma P, Shi J, Munugalavadla V, Sims E, Wei L, Vemula S, Nabinger SC, Goodwin CB, Chan RJ, Traina F, Visconte V, Tiu RV, Lewis TA, Stern AM (2011). Rho kinase regulates the survival and transformation of cells bearing oncogenic forms of KIT, FLT3, and BCR-ABL. Cancer cell.

[77] Martin H, Mali RS, Ma P, Chatterjee A, Ramdas B, Sims E, Munugalavadla V, Ghosh J, Mattingly RR, Visconte V, Tiu RV, Vlaar CP, Dharmawardhane S, Kapur R (2013). Pak and Rac GTPases promote oncogenic KIT-induced neoplasms. The Journal of clinical investigation.

[78] Chatterjee A, Ghosh J, Ramdas B, Mali RS, Martin H, Kobayashi M, Vemula S, Canela VH, Waskow ER, Visconte V, Tiu RV, Smith CC, Shah N, Bunting KD, Boswell HS, Liu Y (2014). Regulation of Stat5 by FAK and PAK1 in Oncogenic FLT3- and KIT-Driven Leukemogenesis. Cell Rep.

[79] Hoermann G, Cerny-Reiterer S, Perne A, Klauser M, Hoetzenecker K, Klein K, Mullauer L, Groger M, Nijman SM, Klepetko W, Valent P, Mayerhofer M (2011). Identification of oncostatin M as a STAT5-dependent mediator of bone marrow remodeling in KIT D816V-positive systemic mastocytosis. The American journal of pathology.

[80] Lee YN, Brandal S, Noel P, Wentzel E, Mendell JT, McDevitt MA, Kapur R, Carter M, Metcalfe DD, Takemoto CM (2011). KIT signaling regulates MITF expression through miRNAs in normal and malignant mast cell proliferation. Blood.

[81] Frost MJ, Ferrao PT, Hughes TP, Ashman LK (2002). Juxtamembrane mutant V560GKit is more sensitive to Imatinib (STI571) compared with wild-type c-kit whereas the kinase domain mutant D816VKit is resistant. Mol Cancer Ther.

[82] Akin C, Fumo G, Yavuz AS, Lipsky PE, Neckers L, Metcalfe DD (2004). A novel form of mastocytosis associated with a transmembrane c-kit mutation and response to imatinib. Blood.

[83] Alvarez-Twose I, Gonzalez P, Morgado JM, Jara-Acevedo M, Sanchez-Munoz L, Matito A, Mollejo M, Orfao A, Escribano L (2012). Complete response after imatinib mesylate therapy in a patient with well-differentiated systemic mastocytosis. J Clin Oncol.

[84] Georgin-Lavialle S, Lhermitte L, Suarez F, Yang Y, Letard S, Hanssens K, Feger F, Renand A, Brouze C, Canioni D, Asnafi V, Chandesris MO, Aouba A, Gineste P, Macintyre E, Mansfield CD (2012). Mast cell leukemia: identification of a new c-Kit mutation, dup(501–502), and response to masitinib, a c-Kit tyrosine kinase inhibitor. Eur J Haematol.

[85] Aichberger KJ, Sperr WR, Gleixner KV, Kretschmer A, Valent P (2008). Treatment responses to cladribine and dasatinib in rapidly progressing aggressive mastocytosis. Eur J Clin Invest.

[86] Gani OA, Engh RA (2010). Protein kinase inhibition of clinically important staurosporine analogues. Nat Prod Rep.

[87] Ghanim V, Herrmann H, Heller G, Peter B, Hadzijusufovic E, Blatt K, Schuch K, Cerny-Reiterer S, Mirkina I, Karlic H, Pickl WF, Zochbauer-Muller S, Valent P (2012). 5-azacytidine and decitabine exert proapoptotic effects on neoplastic mast cells: role of FAS-demethylation and FAS re-expression, and synergism with FAS-ligand. Blood.

[88] Li Z, Cai X, Cai CL, Wang J, Zhang W, Petersen BE, Yang FC, Xu M (2011). Deletion of Tet2 in mice leads to dysregulated hematopoietic stem cells and subsequent development of myeloid malignancies. Blood.

[89] Bai Y, Bandara G, Ching Chan E, Maric I, Simakova O, Bandara SN, Lu WP, Wise SC, Flynn DL, Metcalfe DD, Gilfillan AM, Wilson TM (2013). Targeting the KIT activating switch control pocket: a novel mechanism to inhibit neoplastic mast cell proliferation and mast cell activation. Leukemia.

[90] Lee S, Lee H, Kim J, Lee S, Kim SJ, Choi BS, Hong SS, Hong S (2014). Development and biological evaluation of potent and selective c-KIT(D816V) inhibitors. Journal of medicinal chemistry.

